# Cancer in Japan

**Published:** 1887-09

**Authors:** 


### *CANCER IN JAPAN.*

Through the kindness of Dr. Cushing, we received a reprint of "Contributions to Surgery," by Dr. Hashimoto, Imperial Surgeon to the Japanese Army in Tokio, which were published originally in Langenbeck's Archives. The contributions cover a large field, and contain much valuable personal experience.

We were particularly interested in the contribution to the treatment of carcinomata and sarcomata. Careful scientific observations on these malignant growths in a people whose social habits are so very different from our own, for the mass of the people in Japan still retain their old habits and modes of living, are very interesting from an etiological standpoint. Carcinoma of the breast is of very frequent occurrence among Japanese women, (Dr. Hashimoto having operated on about forty cases during seven years,) and this disease from the most ancient times has been treated by operative procedures in Japan.

The frequency of carcinoma of the breast in Japan is very interesting, because of the difference of dress between the European and the Japanese women. The Japanese women do not wear corsets, or any article of dress which would be apt to irritate the breasts. They wear a broad band of soft cloth or silk around the body below the breasts, tied behind in a large bow-knot.

Carcinoma of the uterus is also very frequent, but patients usually seek the surgeon's advice too late to permit of any other than palliative treatment.

Carcinoma of the tongue is common, but of the lip rare. In reference to the theory of the initiation of the pipe causing carcinoma of the lip, he says that the common people in Japan hold the pipe in a different way from the Europeans. They have a pipe with a very long stem, from which they take a few whiffs, and then lay it aside to be again resumed after a short interval.

Carcinoma of the gullet is common, especially among "sake" drinkers. "Sake" is a kind of alcoholic beverage made from rice, which tastes somewhat like sherry. It is very difficult to understand how such a mild liquor should give rise to cancer. The Japanese for "cancer of the gullet" is "*kak*," and signifies no longer able to eat rice. The *exitus lethelis* occurs about eight or ten months after the disease has been first noticed.

We are indebted to Dr. Cushing for our information about the customs of the Japanese.—*Pacific Medical Journal.*

